# Correction: Phosphorylation of p66Shc and forkhead proteins mediates Aβ toxicity

**DOI:** 10.1083/jcb.20041004106232025c

**Published:** 2025-07-02

**Authors:** Wanli W. Smith, Darrell D. Norton, Myriam Gorospe, Haibing Jiang, Shino Nemoto, Nikki J. Holbrook, Toren Finkel, John W. Kusiak

Vol. 169, No. 2 | https://doi.org/10.1083/jcb.200410041 | April 18, 2005

It was recently discovered that the anti-Shc66 blot in Fig. 5 A (lower panel blot) had been duplicated in Fig. 1 B (lower panel blot). The authors have reviewed the records of the original western blots from this study and discovered that, due to the similarity in the cropped blots, the wrong image was inadvertently chosen for Fig. 1 B. The authors have located the correct blot image for this figure, and it has now been replaced in the paper. The conclusions of the paper are not affected by this error. The error appears in print and in PDFs downloaded on or before June 20, 2025. The authors apologize for the error and for any confusion this may have caused.

**Figure:**
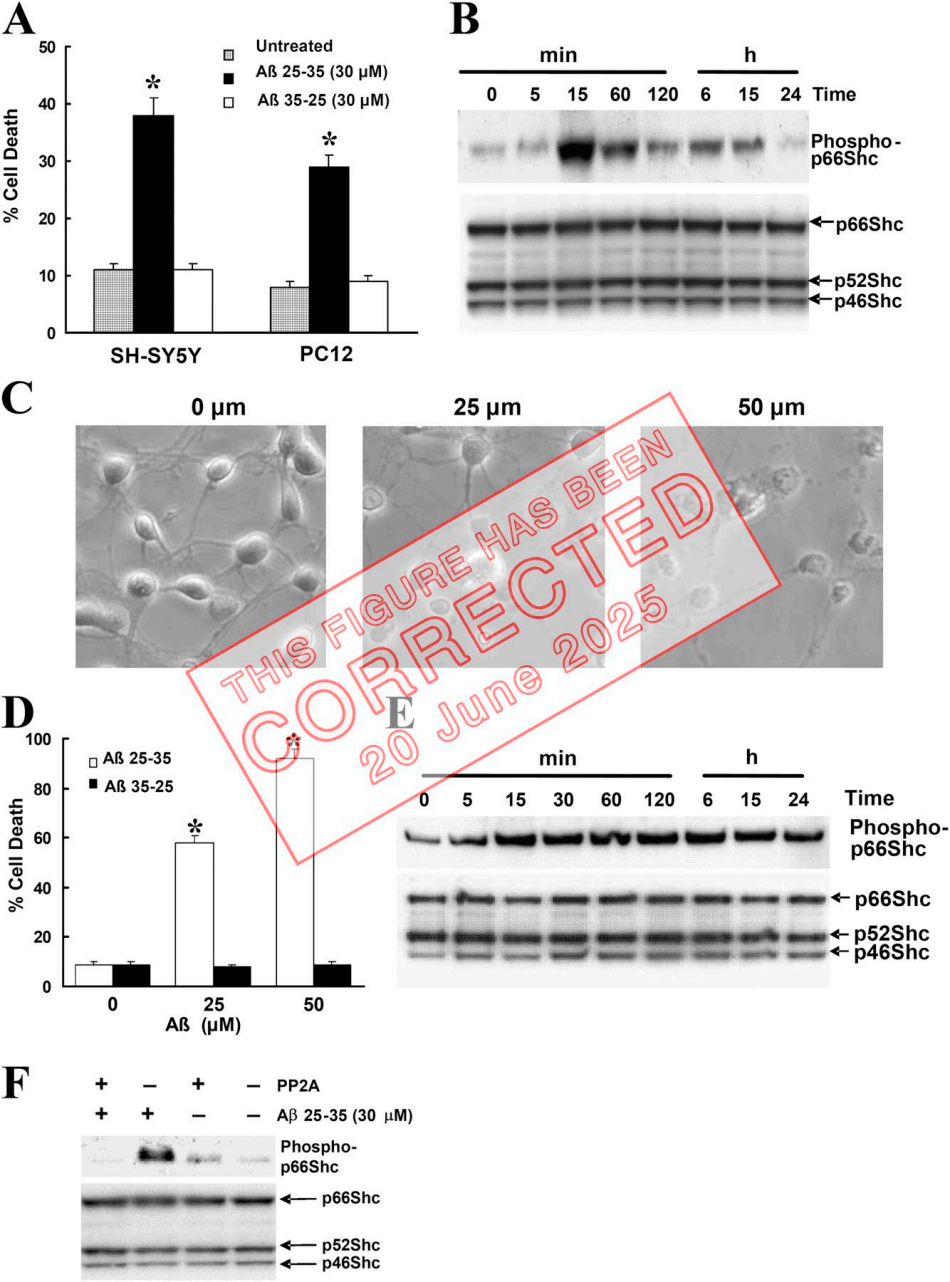


**Figure 1.**
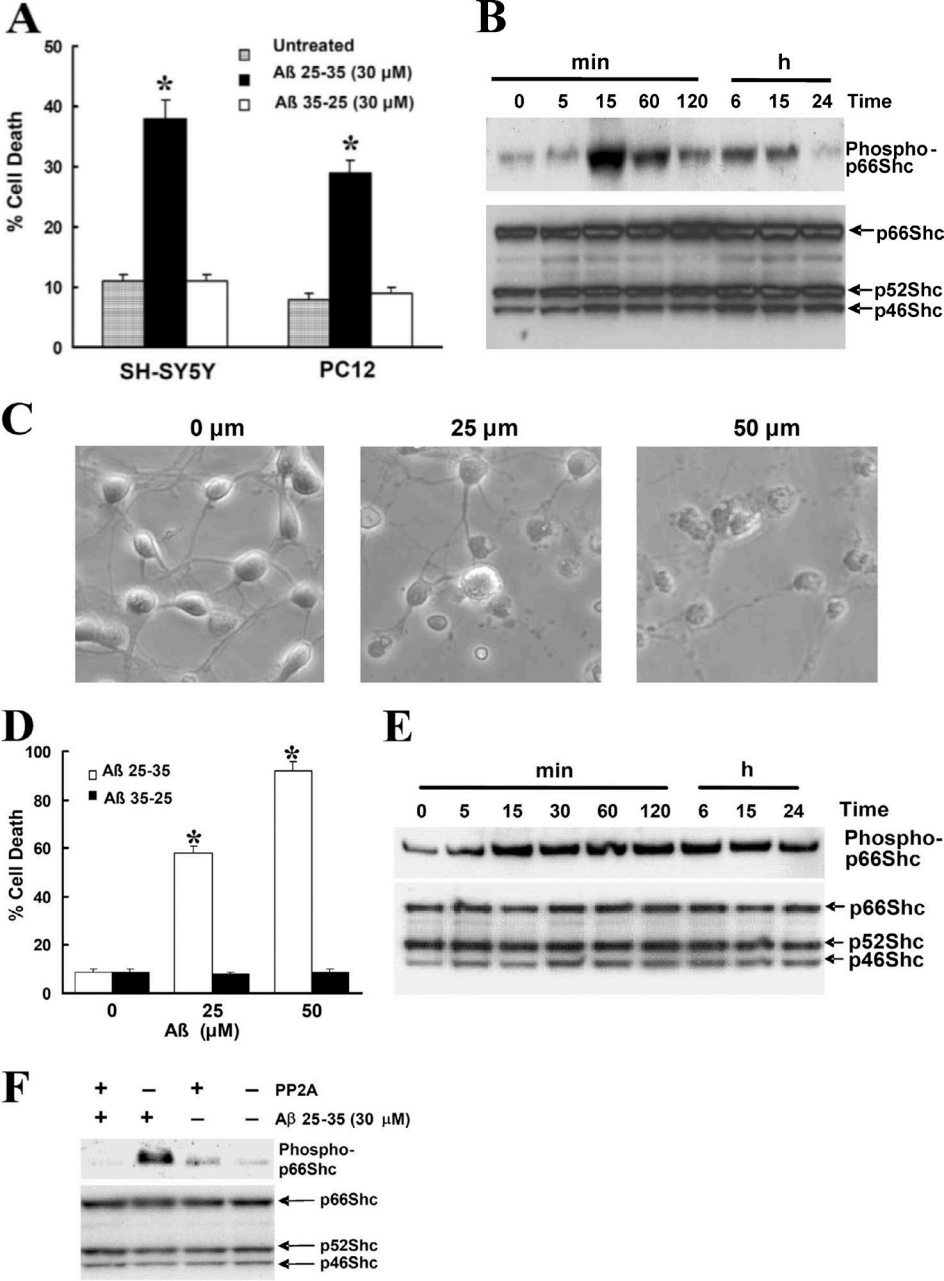
**Treatment of Aβ induces the phosphorylation of p66Shc at Ser36 residues in SH-SY5Y cells. (A)** SH-SY5Y and PC12 cells were treated with 30 µM Aβ25-35 or Aβ35-25 for 24 h in serum-free media containing N2 supplements. Trypan blue exclusion was used to determine cell death. Data are means ± SEM for three separate experiments performed in duplicate. *, P < 0.05 versus untreated cells. **(B)** Western blot analysis showing phosphorylated and total p66Shc in SH-SY5Y cells treated with 30 µM Aβ for the indicated times after a 16-h serum starvation period by using a specific anti–phospho-p66Shc (Ser36) antibody (top) and an anti-Shc polyclonal antibody (bottom). The blots are representative of three separate experiments. **(C)** At 7 DIV, cultures were treated with Aβ for 24 h, whereupon representative photographs were taken to illustrate the changes in cell morphology. **(D)** At 7 DIV, cells treated with Aβ25-35 or Aβ35-25 for 24 h and Hoechst 33342 labeling were used to determine cell death. Data are the means ± SEM of three separate experiments performed in duplicate. **(E)** Western blot analysis to detect p66Shc phosphorylation in 7 DIV cultures treated with 25 µM Aβ for the indicated times was performed by using a specific anti–phospho-p66Shc (Ser36) antibody (top) and an anti-Shc polyclonal antibody (bottom). The blots are representative of three separate experiments. **(F)** SH-SY5Y cells treated with 30 µM Aβ for 30 min after a 16-h serum starvation period. The cells were harvested and the lysates were either treated with PP2A1 or left untreated, and then sequentially blotted with a specific anti–phospho-p66Shc (Ser36) antibody (top) and an anti-Shc polyclonal antibody (bottom). The blots are representative of three separate experiments.

